# Supplementary material for: Human- common hippo (Hippopotamus amphibius)-conflict in the Dhidhessa Wildlife Sanctuary and its surrounding, Southwestern Ethiopia
Source: PLoS One. 2024 May 16;19(5):e0303647. doi: 10.1371/journal.pone.0303647 (PMC11098388; doi:10.1371/journal.pone.0303647)
Supplement: S1 Appendix — (DOCX) [file pone.0303647.s001.docx]

**S1: Appendix: Household questionnaire guide for human-hippopotamus conflict**

Questionnaire: This is the questionnaire interview which was used to examine Human-hippopotamus (*Hippopotamus amphibius*)-conflict in the Dhidhessa Wildlife Sanctuary and its surrounding, Southwestern Ethiopia. The questionnaire interview was conducted with those local farmers who live near the vicinity of the Dhidhessa Wildlife Sanctuary. This information is highly confidential.

Thank you in advance!

**Section # 1: Community survey questionnaire**

Name of Interviewer______________________________________________

Name of Village______________

Date_________ Month_____________ Year__________ Signature______________

**I. Background and socio-economic information**

1. Gender: A. Male B. Female

2. Age : A) 18-24 years B) 25-34 years C) 35-50 years D) 51 and above

3. Marital status: - A) married B) Single C) Divorced D) Widowed

4. Level of education: - A) Illiterate B) Primary School C) Secondary School D) College and above

5. Are you the head of the family numbers? A. Yes B. No

6. How many people live in your household: - A) 1-3 B) 4-6 C) 7-9 D) 10 and above

7. How long have you been living in this area? A) 5-10 years B) 11-15 years C) 16-20 years       D) 21and above

8. What is your main source of income? A) Mixed agriculture B) only farming C) only livestock       rearing D)   trading/self employed

9. How many livestock in numbers? A. 1-10 B.11-20 C. 21-30 D. 31 and above

10. Do you have your own farm land? A. Yes B. No

11. If your choice to question 10 is "A" what is the size of your farmland? A. 0.5-2ha B. 3-5 ha C.6-10    ha D. greater than 10 ha

12. What is the distance of your farm land from Dhidhessa River? A. 1 km far apart B. 1.1-2 km far apart  C. 2.1-3 km far apart D.3.1-4 km far apart E. 4.1-5 km far

13. What type of crop do you cultivate on your farm land? A. Maize B. Sorghum C. Sorghum and maize D. Sesame and ground net E. Sugar cane F. Other

**Section #2: Knowledge, attitudes and currents practice**

Please mark each of the following question based on your knowledge, attitudes and current practices. Where 1= Negative (N), 2= positive (P), 3= Neutral

|  | Statements | N | P | Neutral |
| --- | --- | --- | --- | --- |
| 14 | Do you believe hippopotamus is a good resource for the environment? |  |  |  |
| 15 | Is the conservation of hippopotamus is good things? |  |  |  |
| 16 | What do you think if established conservation of hippo in your area? |  |  |  |

**Section #3: Conflict and crop damage**

17. What kind of problems do you face because of wildlife? A. Crop damage B. physical threat C. Human threat. D. Livestock killing or depredation E. Hippo mortality

18. When was the last time hippopotamus damaged or raided your crops?

A. This year B. Last year C. 2–5 years ago D. 5 years ago

19. Of the crops you grow, which do hippopotamus feed on?

A. Maize B. Sorghum C. Sorghum and Maize D. Sugar cane E. sesame and ground net F. Unsure

20. When crop damage from hippopotamus occurs what are the crop stages?

A. Seedlings (1.5 feet from ground or less) B. Intermediate (2–3 feet from ground)

C. Mature (taller than 3 feet, harvestable)

21. How often do/did hippopotamus raid your crops this year/last year (depending on answer to Q.20)? A. Once B. Twice C. Three times D. More than three times

22. How do you know it was hippopotamus that raided your crops?

A. Footprint B. See the hippopotamus C. Hippopotamus dung D. Appearance of damaged vegetation E. Other (specify)

30. What time of day does crop damage from hippopotamus occur?

A. Morning B. Afternoon C. Evening D. Night E. No specific time F. Unsure

31. In the recent five years, have there been any months where hippopotamus agricultural damage has been more common? _______________________

32. Which of the following ideas can be the reason why it is sever in specific season or month?

A. Shortage of food in the forest or buffer zone B. Presence of plenty crops on the farmland C. unfavorable of the weather condition D .attractiveness of farm crops.

33. Have the number of crop raids by hippopotamus on your farm increased, decreased, or

Stayed the same in the last 5 years?

A, Increased B. Decreased C. Stayed the same C. Unsure

34. What are the causes of human-hippo conflict? A .Don’t know B. Human moved into hippo habitat C. Lack of food D. too many hippo

**Section #4: Deterrent Techniques**35. What do you do to keep animals away from your crops? A. Guarding

B. Physical barriers (fencing ) C. Trenching D. Burning fire E. Other

36. What should happen when hippopotamus damage crops? A. Using traditional method B. Shoot them C. Farmers should be compensated D. Other (specify
**Section #5: Guideline for focus group discussion and key informant interviews**

Interviews guide for official experts and managers of Arjo Dhidhessa Sugar Factory, Environmental protection authority, and farmers was interviewed

1. Do you believe hippopotamus is a good resource for the environment?

2. Is there human hippo conflicts?

3. What kind of problems do you face because of human hippo conflict?

4**.** How do you perceive about the following in your district in general and the study area in particular?

- The major factors causing Human hippo conflict?
- The perception of farmers and expertise towards natural resource degradation problems and human hippo conflict
- The consequences of human hippo conflict on  environment, social and economic aspects

5. Describe the different techniques you use to control (minimize) the damage or conflict caused     by hippo. I ......................... ii......................... iii..........................

6. Which of these techniques are?

I. Most effective...................................... II. Least effective........................................................
