# Supplementary material for: Human- common hippo (Hippopotamus amphibius)-conflict in the Dhidhessa Wildlife Sanctuary and its surrounding, Southwestern Ethiopia
Source: PLoS One. 2024 May 16;19(5):e0303647. doi: 10.1371/journal.pone.0303647 (PMC11098388; doi:10.1371/journal.pone.0303647)
Supplement: S2 Appendix — (DOCX) [file pone.0303647.s002.docx]

**S2: Appendix: Supplementary**

**S1 Table: Mode of crop damage, stage and preference by hippopotamus (Seedlings (1.5 feet from ground or less), Intermediate (2–3 feet from ground), Mature (taller than 3 feet)**

| Types of crops | Mode of damage | Seedling | Intermediate | Mature | Preference |
| --- | --- | --- | --- | --- | --- |
| Maize | Feeding and trampling | NO | ✓ | ✓ | 1^st^ |
| Sugar cane | Feeding and trampling | NO | ✓ | ✓ | 2^nd^ |
| Sorghum | Feeding and trampling | NO | ✓ | ✓ | 3^rd^ |
| sesame | Feeding and trampling | NO | ✓ | ✓ | 4^th^ |
| Ground net | Feeding and trampling | NO | ✓ | ✓ | 5^th^ |
| Banana | Trampling | ✓ | ✓ | ✓ | 6^th^ |
| Onion | Feeding and trampling | ✓ | ✓ | ✓ | 7^th^ |
| Rice | Feeding and trampling | NO | ✓ | ✓ | 8^th^ |


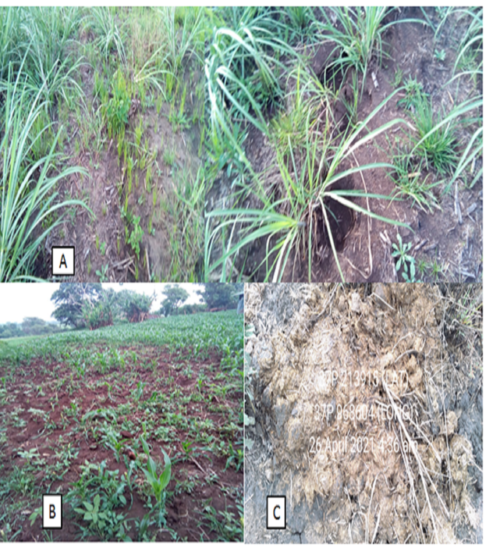


**S1 Fig: Evidence for crops damaged by hippopotamus: (a) sugar, (b) maize, and (c) dung of hippo in the study area**


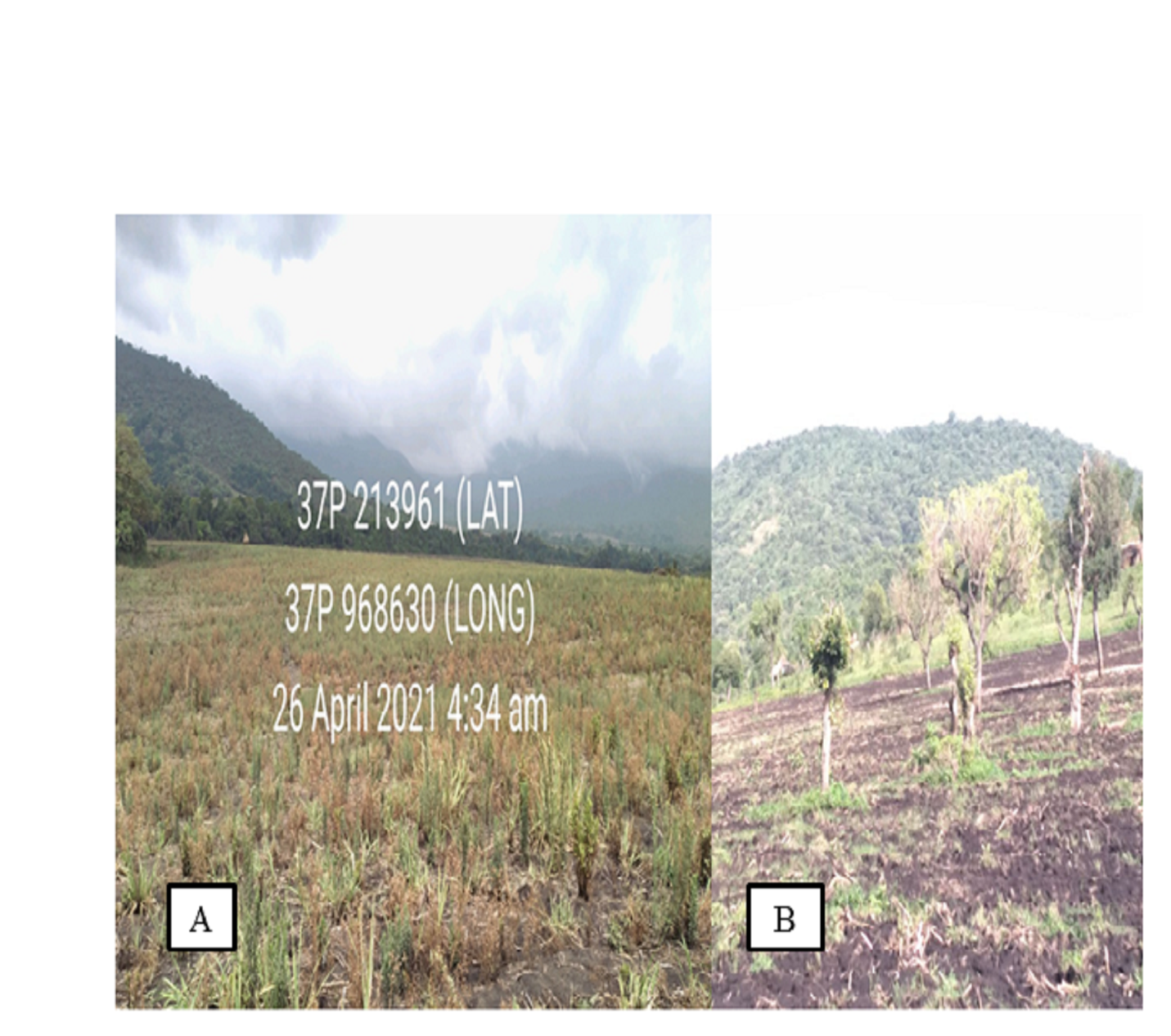


**S2 Fig: Habitat disturbance (A and B) by subsistence agriculture in the study area**


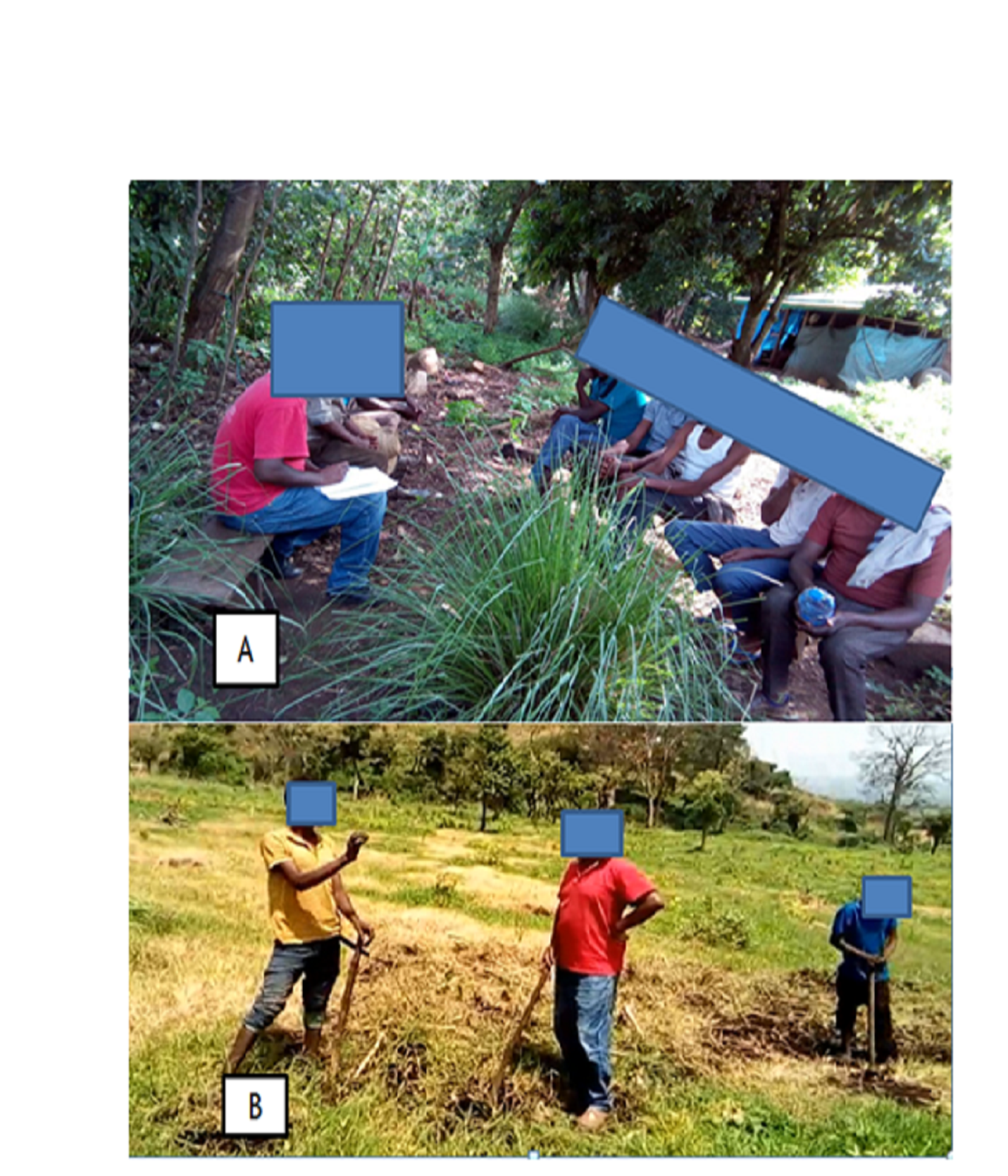
 **S 3 Fig: (a) Focus group discussions and (b) interviewer person with local residents in the study area**


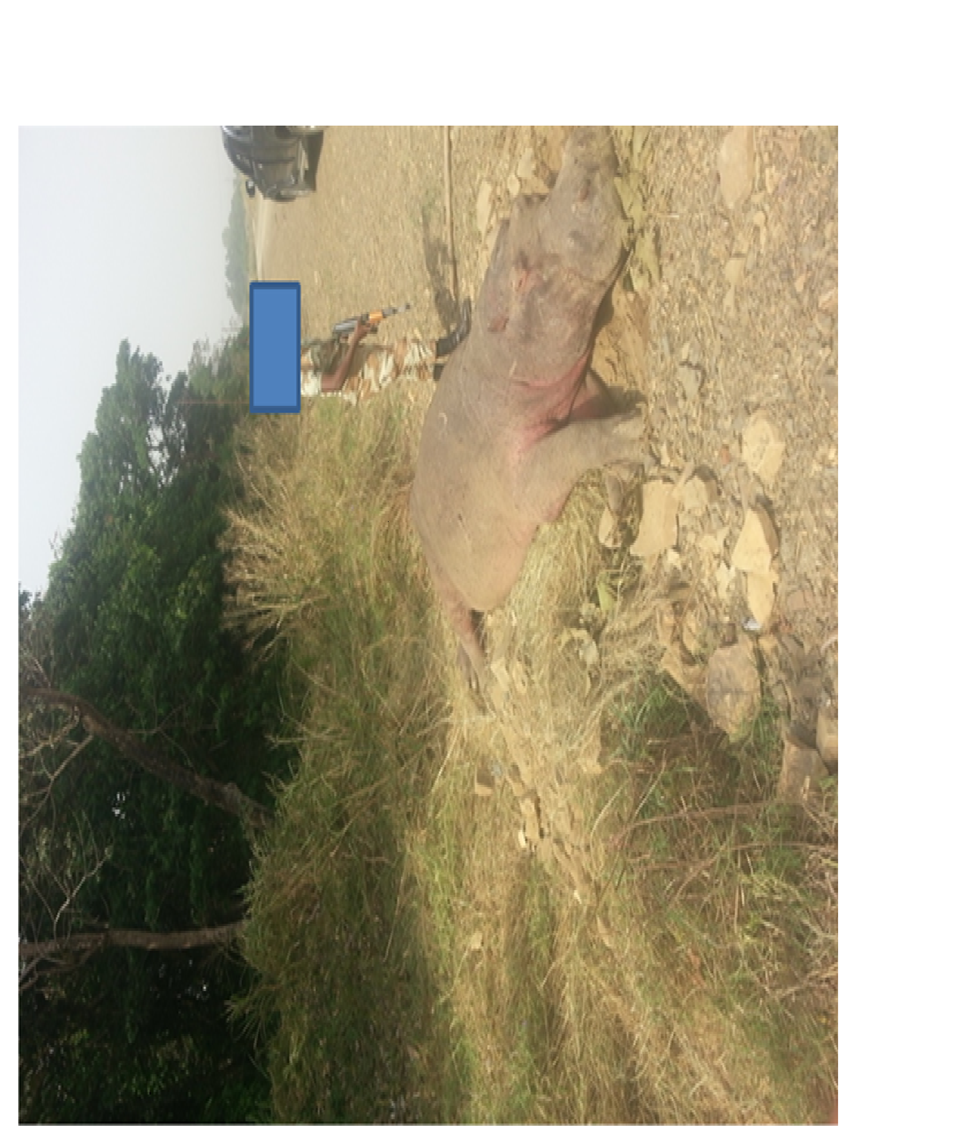
**S 4 Fig: Dead hippos by military around Dhidhessa River in the study area**
