# Supplementary material for: Human- common hippo (Hippopotamus amphibius)-conflict in the Dhidhessa Wildlife Sanctuary and its surrounding, Southwestern Ethiopia
Source: PLoS One. 2024 May 16;19(5):e0303647. doi: 10.1371/journal.pone.0303647 (PMC11098388; doi:10.1371/journal.pone.0303647)
Supplement: Attachment — Submitted filename: renamed_5899c.docx [file pone.0303647.s004.docx]

**To: Stephanie S. Romanach, Ph.D.**

**Academic Editor**

**Journal of PLOS ONE**

**Subject: Responses to the reviewers’ comments**

**Dear Dr. Stephanie S. Romanach,**

We are very grateful for your and reviewers’ critical and constructive comments regarding our manuscript numbered **PONE-D-23-34333**, entitled *“Human-common hippopotamus (Hippopotamus amphibius)-conflict in the Dhidhessa Wildlife Sanctuary and its surrounding, Southwestern Ethiopia”.* Please find re-submitted (the revised version) of our manuscript in which we have carefully considered and addressed all comments raised by the referees. The revised version of the manuscript includes many improvements and summarized below. Moreover, the revised manuscript is presented using track-change with red color. We hope our responses are satisfactory and you will find this version suitable for publication in Journal of PLOS ONE. We look forward to hearing from you.

Dear Academic Editor, please note the following points

1. Our responses to the reviewers’ comments are provided
2. The line numbers with red colors (*line#)* refer to the improved version of the revised manuscript

Best regards,

Corresponding author

**Response to Academic editor #1**

1. **Comment 1(General comment),** the reviewers suggested that the manuscript be corrected for problems of language usage, spelling, grammar, data collection methods and analysis and I found many such problems that were not addressed in the revision.  Some of the revisions introduced new problems as well.  To help improve the quality of the manuscript, I include a list of specific revisions that are needed below.

***Response*:** Thank you very much for your suggestion. We agree with the comment and the whole section is improved by reducing or amending some of the words, phrases or sometimes sentences.

- The manuscript should meet PLOS One's style requirements.
- Manuscript for language usage, spelling, and grammar.
- Grant information in the ‘Funding Information’ and ‘Financial Disclosure’ sections does not match.
- Data availability by the authors upon reasonable request.
- Figures 1 and 2 in your submission contain [map/satellite] images, which may be copyrighted. We made improvements to the issues mentioned above.
  2. **Comment 2,** **Line 1:** Consider replacing with “Human-hippo (Hippopotamus amphibius) conflict..."

**Line 30:** What about rhinos? I would rephrase to say "…one of the most  endangered mega herbivores in Africa…"
  **Line 67:** “…listed as vulnerable…” instead of “tagged vulnerable”

***Response*:** Thank you very much for your suggestions. We agree with the comment and made improvements on **Line 1:** Two endemic species of hippos that are unique to Africa are the common and pygmy hippos, both of which are members of the Hippopotamidae family. Our research thus focused on the common hippo.

**Line 30:** "currently tagged" changed to” one of the most endangered mega herbivores in Africa" and **Line 67:** “tagged vulnerable” changed to “listed as vulnerable” Using a track change from *line# 1, 29 &67*, respectively

3. **Comment 3,** **Line 88-92:** “Unlike most areas in Ethiopia, the culture and actors of HWC in the Dhidhesa wildlife sanctuary is different. Like most hippos dominated human encroached ecosystem, there are plenty of reports for the existence and severity of human-hippo conflict in the area. However, there is no information regarding the causes, extent, and consequences of HHC on both actors in the area are not known. “This paragraph requires rewording; it is a challenge to decipher what the authors are trying to say“

***Response*:** Thank you very much for your suggestions. We agree with the comment and made correction using a track change *from Line# 89-92*; “Recently, Dhidhessa Wildlife Sanctuary has not provided the intended objectives for which it was established. Subsequently, large mammals that occurred in the area migrated into the nearby forest patches. But the hippo is one of many large mammals that have been living in the Dhidhessa River valley. However, in the study area, no comprehensive research has been done on the causes, extent, and consequences of HHC in the southwestern regions of Ethiopia“.

4. **Comment 4,** **Line 100-117:** The “Ethical statement” should not be the first paragraph of the Methods Section. This should either come later in the methods or at the very end of the manuscript, towards the acknowledgements section. There also appears to be another “Methods” section below “Materials and Methods” – try be more specific e.g., Study Design or Survey Approach

***Response*:** Thank you very much for your suggestions. But the guidelines of PLOS ONE stated that “ethical statement” should be the first paragraph under methods Section. However, we agree with the comment and made correction “Materials and Methods” We specified to “Site selection and sampling design” *from Line# 142*

5. **Comment 5**, **Line 133-135:** Not clear whether the reserve was designated for its high diversity or large hippo populations? This needs to be made clearer.

***Response*:** Thank you very much for your comments/suggestions. We agree with the comment and made correction by rewriting the paragraph using a track change *from Line# 133-141:*” Even so, the area is not officially protected at the moment because it is heavily utilized by the private sector and local communities for mechanized monoculture cane farms, subsistence farming, husbandry, and other economic activities that put them in direct conflict with the hippo and other wildlife species in the area. Nevertheless, it remains an important area for biodiversity conservation in the southwestern parts of Ethiopia. In addition to hippos, DWS and the adjacent surroundings are home to a diversity of animal species, including primates (*Papio anubis* and *Cercopithecus mitis*), Buffalo (*Syncerus caffer)*, Lion (*Panthera Leo*) Guerza (*Colobus guerza*),Warthog (Phacochoerus africanus), Bushpig (*Potamochaerus larvatus*) and Spotted Hyena (*Crocuta crocuta*) [ 20]”

6. **Comment 6, Line 142**: “…human-dominated ecosystem…” rather than “…human ecosystem…”

***Response*:** Thank you very much for your suggestions. We agree with the comment and made correction rewrite using a track change *from Line# 133-141*

7. **Comment 7,** **line 157:**   “Focus group discussions” do not need to be abbreviated if it is only mentioned a handful of times in the manuscript, but if it is abbreviated, then be sure to abbreviate as FDG when this is first used, and then use the abbreviation somewhere in the main text.
   **Line 159:** “…total HUMAN populations…”? Unclear what population the authors are referring to.
   **Line 162-164:** remove, “…for the randomization…” as the randomization idea is mentioned again in the same sentence
   **Line 165:** It would be helpful to briefly describe what a semi-structured questionnaire is?
   **Line 168:** “…utilized in the area…” rather than “…experienced…”

***Response*:** Thank you very much for your comments. We agree with the comment and made correction *from Line#* 157,159,162-164,165 and 168 “As you indicated, the data collection methods and analysis paragraph are organised poorly." So, based on your comments and suggestions using a track change *from* *Line#* 163-207, we re-write or revised the whole of those portions.

8. **Comment 8,** **Line 170:** What is the Kobo Toolbox? This needs to be explained after is introduced to the reader. Include a sentence of what this is and why it was used.

***Response*:** Thank you very much for your comments and suggestions. We agree with the comment and made correction using a track change *from* *Line#* 172-174. As you indicated, KoBoToolbox is a suite of tools for field data collection for use in remote areas or where the internet facility is uncertain. Moreover, it is a very easy tool to use and copy the stored data (https://support.kobotoolbox.org/).

9. **Comment 9, Line 183:** Describe which program was used to conduct the analysis only at the end of the paragraph.

***Response*:** Thank you very much for your comments. We agree with the comment and made correction using a track change *from* *Line#* 216-217 (we added at the end of the paragraph)

***10.* Comment 10,** **Line 184-186:** Alternatively, “…To determine the effect of distance, village and perception on HHC…” etc.

***Response*:** Thank you very much for your comments and suggestions. We agree with the comment and made correction using a track change *from* *Line#* 209-217 (as indicated that, we rewriting the analysis section)

11. **Comment 11, Line 192:** 43% is not “most”. Alternatively, “Less than half of the communities have a primary level of education (43%).”?

***Response*:** Thank you very much for your suggestions. We agree with the comment and made correction. We rewrite the demography and Socio-economic activities *from* *Line#* 220-229 with track changes.

12. **Comment 12,** **Line 252-273:** While this paragraph is insightful, it needs to be reworded to be less speculative. The authors could state that “…of the people interviewed, there was a strong trend of increased conflict from 2014, which so happens to coincide with encroachment onto their habitat…”. Or whatever makes most sense from the data available.

***Response*:** Thank you very much for your comments and suggestions. We agree with the comment and made correction using a track change *from* *Line#* 296-320 (this section was reworded by using the data of the interviewee, as suggested)

13. **Comment 13,** **Line 303:** “Refrain from using the word “proved” and instead use “…we show…”or“…our data show…”
    ***Response*:** Thank you very much for your comments. We agree with the comment and made correction using a track change *from* *Line#* 350
14. **Comment 14,** **Line 304-307:** An insightful finding from the study, but again it needs to be re-written and re-worded to make it understandable.

***Response*:** Thank you very much for your comment. We agree with the comment and made correction using a track change *from* *Line#* 352-357 ("involving" re-write and re-worded as suggested).

15. **Comment 15,** **Line 319-320:** Do you mean that the population size of hippos in the DWS is higher than reported elsewhere in Africa?

***Response:*** Thank you very much for your comment. We agree with the comment and made correction using a track change *from* *Line#* 369-372 (replaced by: “This demonstrated that there was a high incidence of crop raiding in the study area, which is dependent on a number of factors like the level of human activity on the farm, the accessibility of resources for hippo, and the cultivation of crops in relation to the sanctuary location”).

16. **Comment 16,** **Line 336:** An important reference to be included here and elsewhere in the manuscript: Van Houdt, S. & Traill, L.W. (2022). A synthesis of human conflict with an African mega herbivore; the common hippopotamus. Frontiers in Conservation Science 3, 954722.
    ***Response*:** Thank you very much for your comments and suggestions. We agree with the comment and made correction using a track change *from* *Line#* 390, 442 and 463
17. **Comment 17,** **Line 372-376:** Which is a strong motivating factor to improve access to education for young woman in the community?

***Response*:** Thank you very much for your suggestions. We agree with the comment and made correction using a track change *from* *Line#* 429-435 ("The results of the study indicate that young women in the study area were driven by a variety of factors to improve access to education, including independence, the ability to make their own decisions, empowerment, employment, and the benefits of natural resources, in order to support their current and future families").

18. **Comment 18,** **Line 424:** Reference: Voysey et al (2023). Are hippos Africa’s most influential mega herbivore? A review of ecosystem engineering by the semi-aquatic common hippopotamus. Biological Reviews

***Response*:** Thank you very much for your suggestions. We agree with the comment and made correction using a track change *from* *Line#* 487

19. **Comment 19,** **Table 1:** It is unclear what “With Farms” and “Without Farms” means here? Describe what this means in the table heading.

***Response*:** Thank you very much for your comment. We agree with the comment and made correction. We describe those values “with farms “mean having cultivated crops, while values “without farms “mean non-cultivated crops.

20. **Comment 20*,*** **Figure 4:** Does this figure describe to what extent the respondents are in support of hippo conservation? It is not perfectly clear that this is indeed the case.

***Response*:** Thank you very much for your suggestions. We agree with the comment and made correction using a track change *from* *Line#* 265-271,272-287 ("People’s perceptions and attitudes towards hippo conservation under the analysis section were rewritten, and figure 4 was removed and replaced by table 4”).
