# Supplementary material for: Human- common hippo (Hippopotamus amphibius)-conflict in the Dhidhessa Wildlife Sanctuary and its surrounding, Southwestern Ethiopia
Source: PLoS One. 2024 May 16;19(5):e0303647. doi: 10.1371/journal.pone.0303647 (PMC11098388; doi:10.1371/journal.pone.0303647)
Supplement: Attachment — Submitted filename: renamed_f1415.docx [file pone.0303647.s005.docx]

**To: Stephanie S. Romanach, Ph.D.**

**Academic Editor**

**Journal of PLOS ONE**

**Subject: Responses to the reviewers’ comments**

**Dear Dr. Stephanie S. Romanach,**

We are very grateful for your and reviewers’ critical and constructive comments regarding our manuscript numbered **PONE-D-23-34333**, entitled *“Human-common hippo (Hippopotamus amphibius)-conflict in the Dhidhessa Wildlife Sanctuary and its surrounding, Southwestern Ethiopia”.* Please find re-submitted (the revised version) of our manuscript in which we have carefully considered and addressed all comments raised by the referees. The revised manuscript is presented using track-change with red color and we attached also the manuscript without track. We hope our responses are satisfactory and you will find this version suitable for publication in Journal of PLOS ONE. We look forward to hearing from you.

Dear Academic Editor, please note the following points

1. Our responses to the reviewers’ comments are provided
2. The line numbers with red colors (*line#)* refer to the improved version of the revised manuscript

Best regards,

Corresponding author

**Response to Academic editor #1**

1. **Comment 1(General comment),** some of the review comments were not addressed in your response. For example, has the statistical analysis been performed appropriately and rigorously?

**Response:** Thank you very much for your comments. We agree with the comment and made correction., Based on the comments and suggestions using a track change from Line# 143-217, 474,480,483,488 and 490 we have re-written or revised the whole of those portions.

- We have rewritten the demography and Socio-economic activities *from* *Line#* 220-229 with track changes
  2. **Comment 2*,*** **Figure 4:** Does this figure describe to what extent the respondents are in support of hippo conservation? It is not perfectly clear that this is indeed the case.

***Response*:** Thank you very much for your suggestions. We have made correction using a track change *from* *Line#* 265-271,272-287 ("People’s perceptions and attitudes towards hippo conservation under the analysis section were rewritten, and figure 4 was removed and replaced by table 4”).

3. **Comment 3,** **Line 185 and 186:** Kebele should be capitalized

***Response*:** Thank you very much for your suggestions. It has been changed as commented, on **Line 185 and 186**.

4. **Comment 4,** **Line 184:** Woreda should also be capitalized

***Response*:** Thank you very much for your suggestions. We have changed as commented, *from Line# 184-186*.

5. **Comment 5,** **Line 209:** change to “analyze”

***Response*:** Thank you very much for your suggestions. We have changed as commented, on Line **209**.

6. **Comment 6**, **Line 215:** What is text analysis? Please describe

***Response*:** Thank you very much for your comments/suggestions. We agree with the comment and made correction by rewriting the words using a track change *from Line# 214-215:”* Information gathered from focus group discussions was analyzed to create a summary before being presented narratively”

7. **Comment 7, Line 236:** do you mean trampled?

***Response*:** Thank you very much for your suggestions. We agree with the comment and made correction to ‘trampled’ as commented on line 236.

8. **Comment 8,** **line 245:**  what percent loss is this? Or out of how many total cattle in the area? ***Response*:** Thank you very much for your comments. We agree with the comment and made correction *from Line#* 245-246 “The results indicated that respondents throughout each surveyed village reared about 2368 cattle. Nearly 27.7% (n = 655) of the cattle from all the villages surveyed died as a result of the fatal infectious disease between 2013 and 2022’’. **Comment 9,** **Line 257:** remove “of responses”

***Response*:** Thank you very much for your comments and suggestions. We have removed as commented *on* *Line#* 258.

9. **Comment 10, Line 260:** remove “from time to time”, suggested revision to “over time”. ***Response*:** Thank you very much for your comments. We have changed as commented *on* *Line#* 261.
10. **Comment 11,** **Line 275:** L 275 and other planes (e.g., L 346) “Hippo” should not be capitalized. Please change throughout.

***Response*:** Thank you very much for your comments and suggestions. We have made correction as commented on *Line#* 275-276 and 347.

11. **Comment 12, Line 281:** change “better” to “more”

***Response*:** Thank you very much for your suggestions. We have made correction on line 282.

12. **Comment 13,** **Line 298:** do you mean “three decades ago”?

***Response*:** Thank you very much for your comments and suggestions. We agree with the comment and made correction using a track change *on* *Line#* 299 (this section was reworded to two decades represent a period of 20 years).

13. **Comment 14,** **Line 351:** river should be lower case, same on L 367
    ***Response*:** Thank you very much for your comments. We have changed as commented on Line*#* 352,368.
14. **Comment 15,** **Line 361:** crop names should be lower case.

***Response*:** Thank you very much for your comment. We agree with the comment and have made correction *on* *Line#* 362.

15. **Comment 16,** **Line 426:** age 5? The 5 may need to be removed or edited.

***Response:*** Thank you very much for your comment. We agree with the comment and made correction on *Line#* 427 (edited by: “citation” [45]).
